# Cocaine/Levamisole-Induced, Skin-Limited ANCA-Associated Vasculitis with Pyoderma Gangrenosum-like Presentation

**DOI:** 10.3390/dermatopathology9030026

**Published:** 2022-06-29

**Authors:** Mirjana Urosevic-Maiwald, Jan-Hendrik B. Hardenberg, Jürg Hafner, Marie-Charlotte Brüggen

**Affiliations:** 1 Hautärzte-Zentrum am Zürisee, 8008 Zurich, Switzerland; maiwald@hautaerzte-zz.ch; 2 Faculty of Medicine Zurich, University Zurich, 8032 Zurich, Switzerland; 3 Department of Dermatology, University Hospital Zurich, 8091 Zurich, Switzerland; jan.hardenberg@charite.de (J.-H.B.H.); juerg.hafner@usz.ch (J.H.); 4 Department of Nephrology and Intensive Care Medicine, Charité—Universitätsmedizin Berlin, 10117 Berlin, Germany; 5 Medical Campus Davos, Swiss Institute of Allergy Research (SIAF), 7265 Davos, Switzerland

**Keywords:** cocaine, levamisole associated autoimmune syndrome, pyoderma gangrenosum

## Abstract

The use of levamisole as the most frequent adulterant of cocaine has merged in previously unknown toxicities, notably a disease entity called cocaine/levamisole-associated autoimmune syndrome (CLAAS). Clinically, CLAAS can manifest with diverse cutaneous and extracutaneous features sharing common laboratory findings (neutropenia, autoantibody patterns). We report the case of a cocaine-abusing female patient with relapsing episodes of painful ulcers, worsening and expanding over a three-year period. The case exhibited all features of a drug-induced, skin-limited, ANCA-associated vasculitis, evolving over time to PG-like findings. In both disease stages, the patient responded well to the cessation of cocaine exposure and systemic glucocorticosteroids. This case demonstrates the continuous nature of cutaneous CLAAS manifestations in a single patient. CLAAS has become a major public health issue in the at-risk group of cocaine users, and clinicians should be alert of this condition when treating cocaine users presenting with single or multiple skin ulcerations.

## 1. Background

Since its first report as an adulterant in 2003 [1], levamisole has become the most commonly used substance to “cut” cocaine [1,2]. Severe toxicities, mostly agranulocytosis, had previously led to the drug’s withdrawal from medical use in humans in the US [1]. The illegal use of levamisole in combination with cocaine has harbored additional, previously unknown health issues [3,4,5,6,7]. The most-discussed entity is the cocaine-/levamisole-associated autoimmune syndrome (CLAAS) [4,8,9]. In the skin, CLAAS manifests with retiform purpura [10,11,12,13] or pyoderma gangrenosum (PG)-like features [14,15,16,17,18,19,20,21]. Inner organs such as the kidney can also be affected [3,22,23]. Serological findings in CLAAS include neutropenia and increased titers of antineutrophil cytoplasmic antibodies (ANCA) with positive cytoplasmic (cANCA) and/or perinuclear (pANCA) staining patterns, and anti-neutrophil elastase (NE)-directed antibodies [5,24], and thus qualifies as a drug-induced, skin-limited, ANCA-associated vasculitis [25]. We report the case of a patient suffering from relapsing CLAAS with changing clinical and histological features over time.

## 2. Case Report

**Figure 1:**
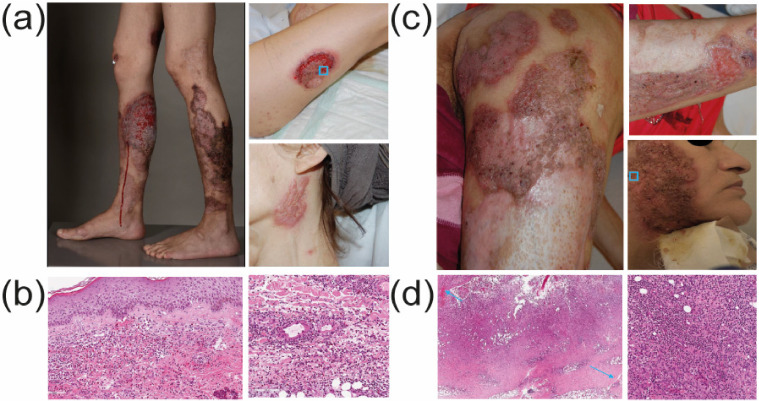
Skin changes and histology of CLAAS over time. (**a**) Clinical presentation upon first admission. The patient had extensive polycyclic ulcerations on lower legs and forearms, partly covered by yellowish exsudate and with an undermined edge and surrounding erythematous–violaceous border. There was also cribriform scarring, as shown on the neck. (**b**) Hematoxylin and eosin (H&E) staining (indicated scale, 100 μm) of a lesional skin biopsy taken from a lesion border (as indicated in (**a**)) shows a leukocytoclastic hemorrhagic vasculitis. There is a massive, subepidermal neutrophilic infiltrate with some rare eosinophils, as well as extensive hemorrhage. (**c**) Clinical presentation after four years. Disseminated cribriform ulcerations and extensive scars on the upper leg (left), forearm and face. (**d**) H&E staining of a lesional skin biopsy taken from an ulcer (as indicated in (**c**)) shows prominent, focally granulomatous neutrophilc infilitrates and fistula in the dermis/hypodermis, which are indicative of PG.

A 57-year-old woman presented at our dermatology outpatient clinic with disseminated painful ulcers. Following their first appearance three years earlier, the ulcers had “come and gone”, but then persisted and expanded over two months. Within this period, the patient also experienced relapsing fevers, night sweats and joint pain. She was reported to consume approximately 1 g of cocaine per day (mostly crack) for several years. Her personal history revealed a non-treated, chronic hepatitis C infection. Dermatological examination showed disseminated polycyclic ulcers covered by yellow exudate and surrounded by prominent red–violet borders (Figure 1a). Our differential diagnosis included granulomatosis with polyangiitis, cocaine-/levamisole-associated vasculitis, PG, ecthymas and superinfected, exogenically inflicted ulcers.

Our diagnostic work-up included blood and urine analyses, wound swabs, a skin biopsy and chest X-ray. The differential blood count showed neutropenia and thrombopenia. Serum p-ANCA titers were increased, as were autoantibodies against NE. Dermatohistopathology (Figure 1b) revealed a leukocytoclastic, hemorrhagic vasculitis with a mostly neutrophilic infiltrate, a direct immunofluorescence was negative. The search for systemic vasculitis involvement was negative. Toxicological urine analyses came back positive for a cocaine metabolite (benzoylecgonine) but not levamisole. The patient tested negative for tuberculosis, HIV and hepatitis B and no relevant infectious agents were found in blood cultures, skin swabs and tissue cultures. We thus retained the diagnosis of a cocaine/levamisole-induced skin-limited ANCA-associated vasculitis in the spectrum of CLAAS.

Our patient agreed to enter a professional drug rehabilitation program (cessation of cocaine use being the mainstay of CLAAS treatment). Topically, we applied daily antiseptic kalium permanganate wound dressings and topical corticosteroids (TCS), namely clobetasol–propionate cream with gaze dressings. The ulcers healed within ca. two weeks and TCS were tapered. The patient was released from the hospital and relapsed on cocaine. She was admitted and treated a few months later for another CLAAS vasculitis episode and was treated with a similar regimen. After three years lost to follow-up, the patient was again admitted for painful ulcers and reduced overall condition. She had disseminated polycyclic ulcers with undermined, purple borders as well as multiple cribriform scars (Figure 1c). Histology revealed massive dermal neutrophilic infiltrates with fistulated areas (Figure 1d), consistent with chronic PG. Serological work-up revealed unchanged autoantibody patterns (increased p-ANCA-, MPO- and NE-antibodies). Toxicological urine analysis was positive for metabolites of heroin, cocaine and levamisole. These findings led us to the diagnosis of multiple PG as a manifestation of CLAAS. We administered oral prednisone (50 mg daily for 2 weeks) with a prescribed tapering over 6 weeks. As a topical treatment, we applied daily antiseptic wound dressings with octenidin solution for ca. 10 min without a subsequent dry dressing. The ulcers healed during the hospital stay within three weeks; the patient has not shown up for the scheduled follow-up control.

## 3. Discussion

In 2010 the first description of a levamisole-associated vasculitis was published [26]. CLAAS is characterized by a distinct serological profile of high titer p-ANCA and positive MPO antibodies in conjunction with purpuric skin lesions [10,23,27]. The distinction between idiopathic ANCA-associated vasculitis and CLAAS can be made via anti-neutrophil elastase antibodies [24]. Typical histological findings are thrombotic vasculopathy and small vessel vasculitis or a mixture of both [10], as we saw in the first dermatohistopathological assessment of our patient. The involvement of inner organs, particularly the kidney, has been described [22,23] but was not present in our case.

The abuse of levamisole-adulterated cocaine has also been linked to PG in a series of cases [16,28]. It was found that these PG patients exhibited the same serological findings as individuals with CLAAS [15]. The association with cocaine/levamisole exposure, the overlap in serological profile and the reoccurring histological findings have prompted the notion that LAC and levamisole-associated PG are clinical variants of an underlying spectrum of CLAAS [9].

The exact pathophysiology behind CLAAS remains elusive. In vitro studies have suggested a role of levamisole-induced NETosis [29]. Levamisole directly causes NETosis through binding to neutrophilic muscarinic type 3 receptors and the resulting NETs were shown to be enriched in neutrophil elastase [29,30]. It is, therefore, conceivable that overshooting NETosis in genetically susceptible individuals leads to autoantibody formation against certain NET associated proteins, such as MPO, PPR3 and NE [9]. It also remains to be elucidated whether and how levamisole/cocaine may impact the disease manifestation and course [16].

The cornerstones of CLAAS treatment are the cessation of levamisole exposure and immunosuppressive treatment [9]. Doses of up to 40 mg oral prednisone have been shown to lead to a quick and marked improvement in skin ulcerations in patients with a PG-like manifestation [15] within CLAAS [10]-. In line with these reports, and despite the impressive and extensive ulcers of our patient, the lesions healed within only weeks of high-potency topical clobetasol propinonate treatment, and, in the second episode, oral course of prednisone at 50 mg per day.

## 4. Conclusions

Our case shows the importance of considering CLAAS as a differential diagnosis for painful ulcers in the at-risk population of cocaine users. Our patient is the first reported case with cutaneous features of CLAAS, i.e., vasculitis and PG, changing over time. Future reports will help to expand our understanding of this dynamic and the means of dealing with it.
